# Absolute quantification of *Mycoplasma pneumoniae* in infected patients by droplet digital PCR to track disease severity and treatment efficacy

**DOI:** 10.3389/fmicb.2023.1177273

**Published:** 2023-06-22

**Authors:** Hanqing Zhao, Chao Yan, Yanling Feng, Bing Du, Junxia Feng, Xiaohu Cui, Jinghua Cui, Lin Gan, Zheng Fan, Ziying Xu, Tongtong Fu, Zihui Yu, Jing Yuan, Guanhua Xue

**Affiliations:** Department of Bacteriology, Capital Institute of Pediatrics, Beijing, China

**Keywords:** *Mycoplasma pneumoniae*, digital droplet PCR, real-time PCR, bacterial load, treatment efficiency

## Abstract

*Mycoplasma pneumoniae* is a common causative pathogen of community-acquired pneumonia. An accurate and sensitive detection method is important for evaluating disease severity and treatment efficacy. Digital droplet PCR (ddPCR) is a competent method enabling the absolute quantification of DNA copy number with high precision and sensitivity. We established ddPCR for *M. pneumoniae* detection, using clinical specimens for validation, and this showed excellent specificity for *M. pneumoniae*. The limit of detection of ddPCR was 2.9 copies/reaction, while that for real-time PCR was 10.8 copies/reaction. In total, 178 clinical samples were used to evaluate the ddPCR assay, which correctly identified and differentiated 80 positive samples, whereas the real-time PCR tested 79 samples as positive. One sample that tested negative in real-time PCR was positive in ddPCR, with a bacterial load of three copies/test. For samples that tested positive in both methods, the cycle threshold of real-time PCR was highly correlated with the copy number of ddPCR. Bacterial loads in patients with severe *M. pneumoniae* pneumonia were significantly higher than those in patients with general *M. pneumoniae* pneumonia. The ddPCR showed that bacterial loads were significantly decreased after macrolide treatment, which could have reflected the treatment efficacy. The proposed ddPCR assay was sensitive and specific for the detection of *M. pneumoniae*. Quantitative monitoring of bacterial load in clinical samples could help clinicians to evaluate treatment efficacy.

## Introduction

*Mycoplasma pneumoniae* is a causative pathogen of respiratory infections in humans. It is a small, cell-wall-deficient bacterium that belongs to the class Mollicutes. *M. pneumoniae* is a significant cause of community-acquired pneumonia, especially in children and young adults (Waites et al., 2017). The clinical manifestations of this pathogen are mostly those of atypical pneumonia, including fever, cough, sore throat, headache, and malaise. Some severe cases have extrapulmonary complications, such as cardiovascular, gastrointestinal, renal, and central nervous system dysfunction (Atkinson et al., 2008). However, the clinical presentations of *M. pneumoniae* are indistinguishable from those of other respiratory pathogens, which also present with fever, cough, and other symptoms of respiratory tract infection. Co-infections, especially with respiratory viruses, are often observed in *M. pneumoniae* infection (Li et al., 2022). Thus, developing a precise and highly sensitive microbiological diagnosis method is important.

As a fastidious bacterium, *M. pneumoniae* is seldom routinely tested for using clinical culture. Thus, alternative molecular methods have been established for *M. pneumoniae* detection, such as real-time PCR, nucleic-acid-sequence-based amplification, loop-mediated isothermal amplification, and recombinase-aided amplification (Diaz and Winchell, 2016). These PCR-based methods all show good analytical detection performance. Real-time PCR is the mainstream diagnostic procedure used in most hospital and reference laboratories (Diaz and Winchell, 2016). The quantitative nature of this method is determining sample concentration by comparing the cycle threshold value (CT) to a standard curve. With regard to precision medicine, the accurate measurement of pathogen-related nucleic acids is becoming more important.

Digital droplet PCR (ddPCR) is an absolute quantification method that does not involve the generation of a standard curve, and it shows robust detection efficiency for various pathogens such as SARS-CoV-2, hepatitis B virus, human immunodeficiency virus, and *Mycobacterium tuberculosis* (Kuypers and Jerome, 2017; Liu et al., 2020). Several studies have shown that ddPCR has greater sensitivity than real-time PCR and is more suitable for samples with a low bacterial load (Taylor et al., 2017). Most importantly, the direct absolute quantification method is useful for evaluating disease progression and treatment efficacy.

In this study, we developed a ddPCR method for the accurate quantification of *M. pneumoniae* and compared its clinical advantages with real-time PCR. We evaluated the ability of the assay to obtain results from samples taken from different periods of *M. pneumoniae* infection, and to measure treatment efficacy based on bacterial load in infected patients.

## Materials and methods

### Clinical specimens and information

In total, 186 clinical respiratory specimens, including 99 sputum samples, 46 throat swabs and 41 bronchoalveolar lavage fluid, were obtained from pediatric pneumonia patients between January and December 2019. Seventy-nine specimens were collected from patients diagnosed with *M. pneumoniae* infection without any co-infection, based on a commercial real-time PCR assay (Mole Bioscience Co. Ltd., Jiangsu, China. target gene P1). The other 99 specimens were negative for *M. pneumoniae* when tested with the same kit. Six patients were sampled two- or three-times during hospital treatment, and these additional eight samples were also used to measure treatment efficacy. Clinical information was also collected, including sex, age, duration of fever, length of stay, white blood cell count, neutrophil ratio, and C-reactive protein level.

All patients were separated into a severe *M. pneumoniae* pneumonia (SMPP) group and a general *M. pneumoniae* pneumonia (GMPP) group based on the guidelines for management of pediatric community-acquired pneumonia (The Subspecialty Group of Respiratory Disease, 2013; Fan et al., 2017). The SMPP group had worsening clinical signs, persistent fever (>38.5°C), and worsening lung imaging (radiological deterioration or consolidation present in more than two-thirds of the lung) and intra- and extrapulmonary complications. The GMPP group showed improvement within 7 days of regular treatment with macrolide antibiotics. This study was approved by the Ethics Committee of the Capital Institute of pediatrics. Informed consent was obtained during admission for patient clinical records to be used in future studies. All data were analyzed anonymously.

### DNA extraction

Total DNA was extracted from 200 μL of the 186 clinical samples using the QIAamp DNA Mini Kit (Qiagen, Hilden, Germany). The extracts were eluted in 150 μL of nuclease-free water and stored at −80°C until use. The concentration of nucleic acids was determined with a NanoDrop 2000 Spectrophotometer (Thermo Scientific, Waltham, MA, United States).

### Principle and workflow of *Mycoplasma pneumoniae* detection with ddPCR assay

**Figure 1:**
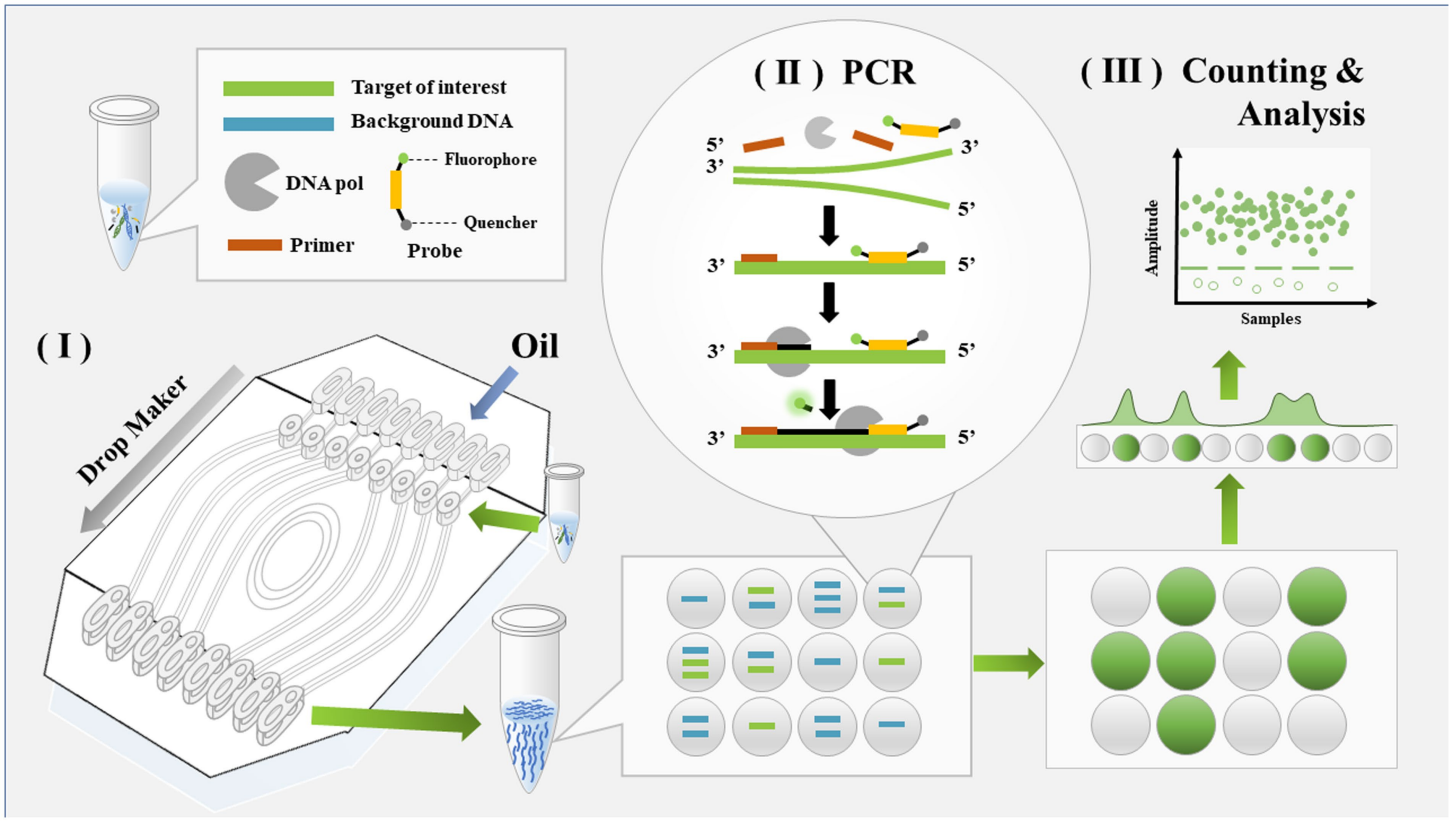
Schematic diagram of detection workflow for *Mycoplasma pneumoniae* using ddPCR. The entire workflow of ddPCR method includes three steps: droplet generation, regular PCR, and droplet analysis.

Digital PCR was conducted by dividing the samples into many droplets and counting the number of droplets in which a reaction occurred. The sample preparation in this assay was the same as for real-time PCR. The workflow of *M. pneumoniae* detection with ddPCR is shown in Figure 1 and included three steps. The samples were first loaded onto a droplet generator and divided into multiple drops. Usually, the number of drops generated was 30,000–60,000. PCR was used to process each drop. After PCR amplification, the fluorescence signal was analyzed with the droplet reader system; in this step, drops containing the target yielded a signal and were assigned as positive, otherwise they were read as negative.

### Digital droplet PCR

Digital droplet PCR was performed with a TargetingOne Digital PCR System (Beijing, China). The primers and probe (Supplementary Table S1) were designed for sequences within the conserved region of the P1 gene (accession no. U00089.2) and BLASTed for *in silico* cross-reactivity. All primers showed high specificity for *M. pneumoniae*. This conserved region was cloned into vector pUC57 to obtain recombinant plasmid for further use. The master mix for the ddPCR was composed of 15 μL of reaction buffer, 6.45 μL of DNase-free water, 2.4 μL of each forward and reverse primer (10 μM), and 0.75 μL of the probe (10 μM) to form a final volume of 20 μL. A no-template control was used in every ddPCR batch. The generation of droplets was performed with a Targeting One Droplet Generator. PCR amplification was carried out using an Applied Biosystems Veriti 96-Well Thermal Cycler with the following conditions: 95°C for 10 min followed by 40 cycles of denaturation at 94°C for 30 s and 60°C for 1 min. The plate was stored at 4°C until the droplets were analyzed with a Targeting One Droplet Reader and Chip Reader R1 software.

### Real-time PCR

Real-time PCR was conducted in parallel with the same primers and probes used for ddPCR on ABI 7500 Software Version 2.3 (Applied Biosystems). The reaction system included 15 μL of PCR Master Mix Reagents (Tiangen Biotech Co. Ltd., Beijing, China), 0.9 μL of forward primer (10 μM), 0.9 μL of reverse primer (10 μM), 0.6 μL of probe (10 μM), and 9.6 μL of DNase-free water, and the same amount of DNA template as was used for ddPCR. The cycling conditions were as follows: 94°C for 10 min, followed by 40 cycles of denaturation at 94°C for 10 s and annealing/extension at 60°C for 45 s. The result was considered positive when the cycle threshold (Ct) for the P1 gene was ≤38 and negative when Ct was >38.

### Analytical sensitivity and specificity of digital droplet PCR

The specificity was evaluated by testing for other *Mycoplasma* and common bacteria of the respiratory tract: *M. pneumoniae* type 1 strain (ATCC 29342), *M. pneumoniae* type 2 strain (ATCC 15531), *Streptococcus pneumoniae* (ATCC 49619), *Haemophilus influenzae* (ATCC 43065), *Staphylococcus aureus* (ATCC 25923), *Staphylococcus epidermidis* (ATCC 12228), *Klebsiella pneumoniae* (ATCC 27736), *Escherichia coli* (ATCC 25922), *Mycoplasma fermentans* (ATCC 19989), *Legionella pneumophila* (ATCC 33152), *Mycobacterium tuberculosis* (ATCC 25618/H37Rv), *Pseudomonas aeruginosa* (ATCC27853), *Mycoplasma genitalium* (ATCC 33530), *Ureaplasma parvum* (ATCC 27813), *Mycoplasma hominis* (ATCC 23114), adenovirus (DNA from clinical isolates), herpes simplex virus (DNA from clinical isolates), and varicella zoster virus (DNA from clinical isolates). The linear dynamic range of the ddPCR assay was determined using 10-fold serial dilutions of the recombinant plasmid. For the reproducibility of ddPCR, five independent experiments were performed with these recombinant plasmid dilutions across 5 days, then the inter-assay coefficient of variance (CV) was calculated. The limit of detection (LoD) of both real-time PCR and ddPCR was obtained using serial dilutions of spiked samples of concentrations close to the detection limits. For all concentrations, tests were performed on 20 replicates.

### Performance of digital droplet PCR and real-time PCR using clinical samples

To evaluate the performance of the ddPCR assay for *M. pneumoniae* detection, 178 clinical samples were tested. The performance was compared with that of real-time PCR in parallel, using the same primers and probes.

### Statistical analysis

Categorical variables (Sex) are expressed as count/percentages, and between-group comparison was done using the Chi-squared test. The distributions of continuous variables were assessed for normality using the Shapiro–Wilk W test. Continuous variables are expressed as mean (standard deviation) in the case of Gaussian distributions or median (interquartile range) in the case of skewed distributions. Comparison of continuous variables between two groups was performed using the independent samples t-test or nonparametric Mann–Whitney U test, where appropriate. Correlations between the Ct values of real-time PCR and the bacterial load determined by ddPCR were analyzed with the Pearson correlation test. A *p* value <0.05 (two sided) was considered statistically significant. The above-mentioned analyses were performed using either Prism 8.0 (GraphPad, La Jolla, CA, United States) or SPSS 19.0 (College Station, TX, United States) software.

## Results

### Analytical performance of the *Mycoplasma pneumoniae* digital droplet PCR assay

**Figure 2:**
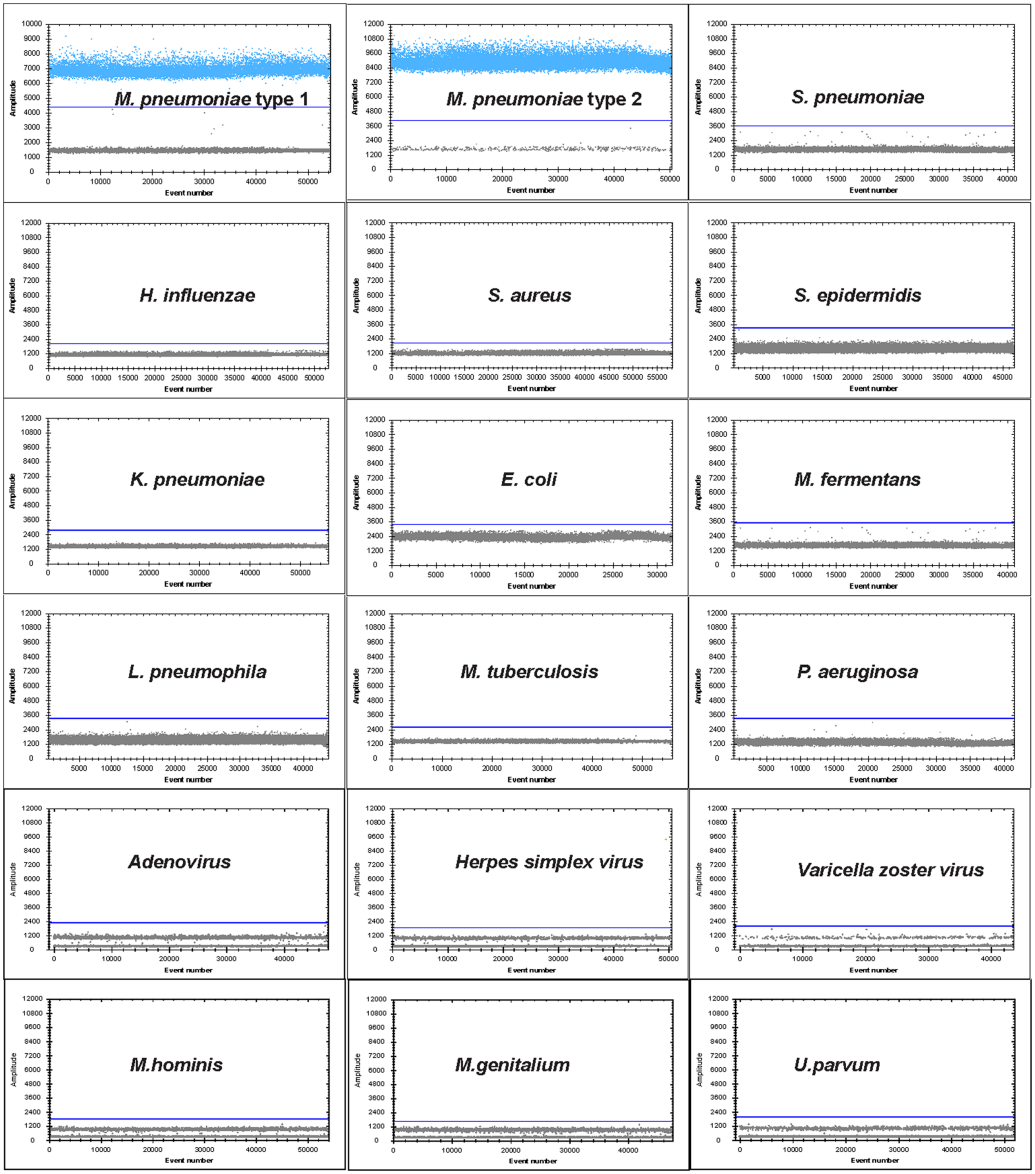
Specificity of ddPCR assay for *M. pneumoniae.* Positive droplets of *M. pneumoniae* with channel amplitude signals above 6,500. The test was negative for the other 12 pathogens of the respiratory tract: *S. pneumoniae*, *H. influenzae*, *S. aureus*, *S. epidermidis*, *K. pneumoniae*, *E. coli*, *L. pneumophila*, *M. tuberculosis*, *P. aeruginosa*, adenovirus, herpes simplex virus, varicella zoster virus and four mollicutes species *M. fermentans*, *M. genitalium*, *U. parvum* and *M. hominis*.

The ddPCR assay showed excellent specificity for *M. pneumoniae* detection. Among the 12 common respiratory pathogen controls and four mollicutes species, only two *M. pneumoniae* samples (types 1 and 2) produced positive signals, while the other control samples were negative. Therefore, ddPCR showed high specificity for *M. pneumoniae*, with no cross-reaction with other pathogens (Figure 2).

**Figure 3:**
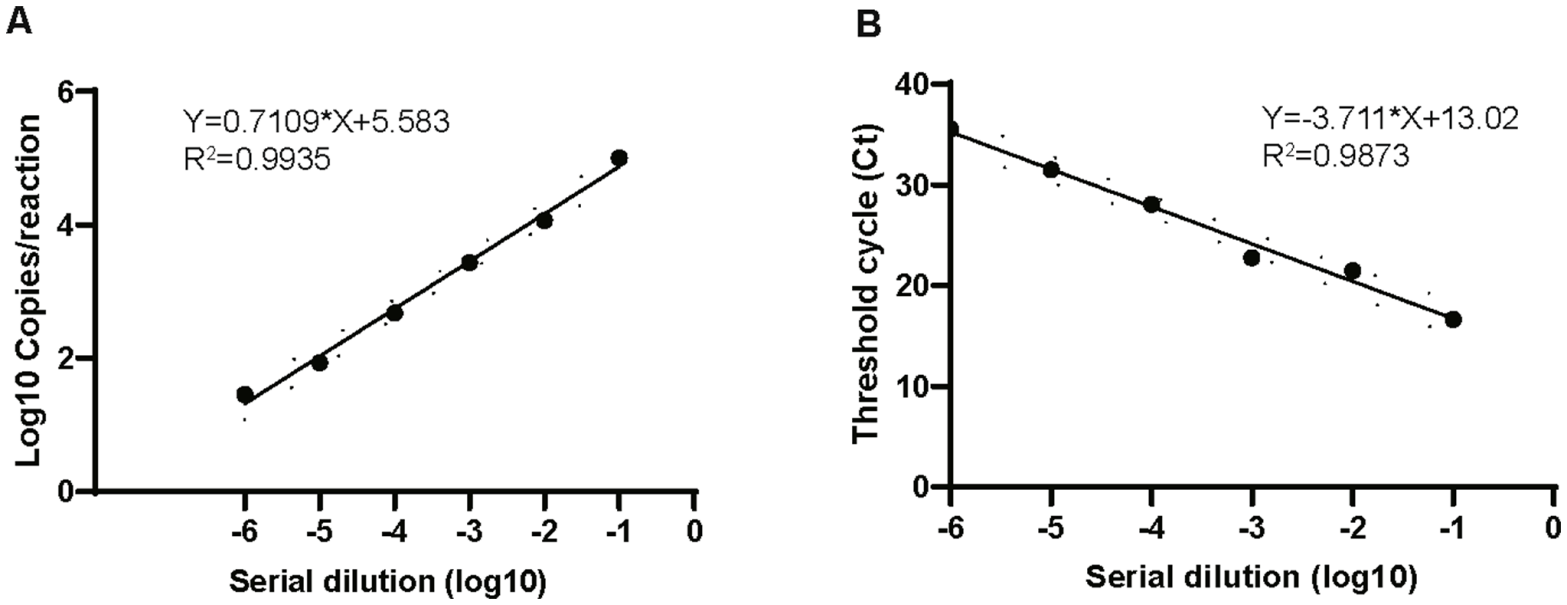
Comparison of dynamic range of ddPCR and real-time PCR. Plots of results from linearity experiment for quantification of *M. pneumoniae* with ddPCR **(A)** and real-time PCR **(B)**. Dilution multiples from 10^6^ to 0 copies/μL are plotted on the *x*-axis. ddPCR (converted to log10) and real-time PCR (Ct) are plotted on the *y*-axis using GraphPad Prism 8.0.

The linear dynamic range was evaluated by ddPCR and compared with that of real-time PCR. Both methods showed a good linear range, and ddPCR achieved results comparable to those of real-time PCR: *R*^2^ for ddPCR was 0.9935 and that for real-time PCR was 0.9873 when testing the 10-fold serial dilutions from 10^5^ to 10 copies/μL of the recombinant plasmids (Figure 3) The inter-assay CV of different plasmid dilutions (10^5^, 10^4^, 10^3^, 100, and 10) were 0.068, 0.094, 0.116, 0.159, and 0.197, respectively, suggesting that the ddPCR assay had good reproducibility.

### LoD of *Mycoplasma pneumoniae* digital droplet PCR and real-time PCR

We used serial dilutions of the recombinant plasmid to determine the detection limit of ddPCR and compared it with that of the real-time PCR assay. The linear series DNA standards were diluted to the detection limit with cDNA from negative throat swab samples from healthy people to concentrations below the minimum detection range of ddPCR or real-time PCR. Each concentration was analyzed for 20 replicates. The LoD of ddPCR was 2.9 (95% CI: 1.8–4.4) copies/reaction while the real-time PCR was 10.8 (95% CI: 7.1–14.5) copies/reaction. The LoD of ddPCR and real-time PCR were comparable, ddPCR was probably three times below that of the real-time PCR.

### Evaluation of the performance of digital droplet PCR and RT-PCR for clinical samples

**Figure 4:**
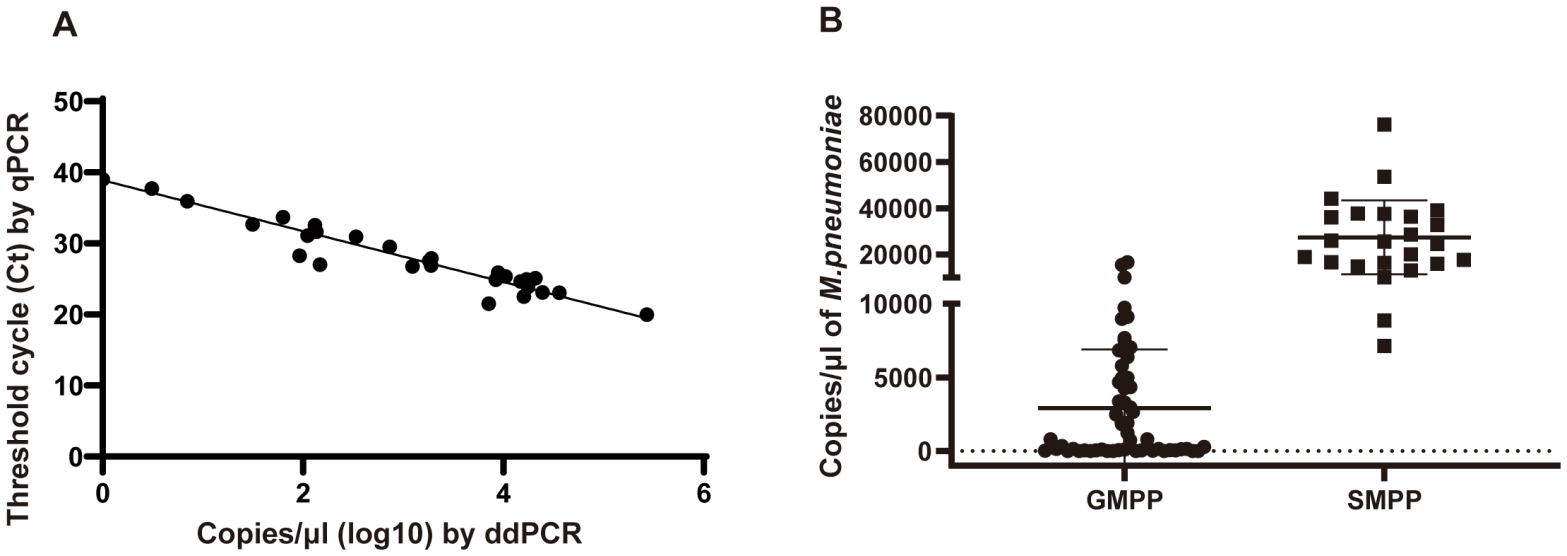
Performance of ddPCR in clinical samples. **(A)** Correlation analysis between ddPCR copies and Ct values of real-time PCR. Copies of ddPCR (converted to log10) are plotted on the *x*-axis. Ct values of real-time PCR are plotted on the y-axis using GraphPad Prism 8.0. **(B)** The copy numbers of *M. pneumoniae* positive patients in different groups. SMPP, severe *M. pneumoniae* pneumonia. GMPP, general *M. pneumoniae* pneumonia.

**Figure 5:**
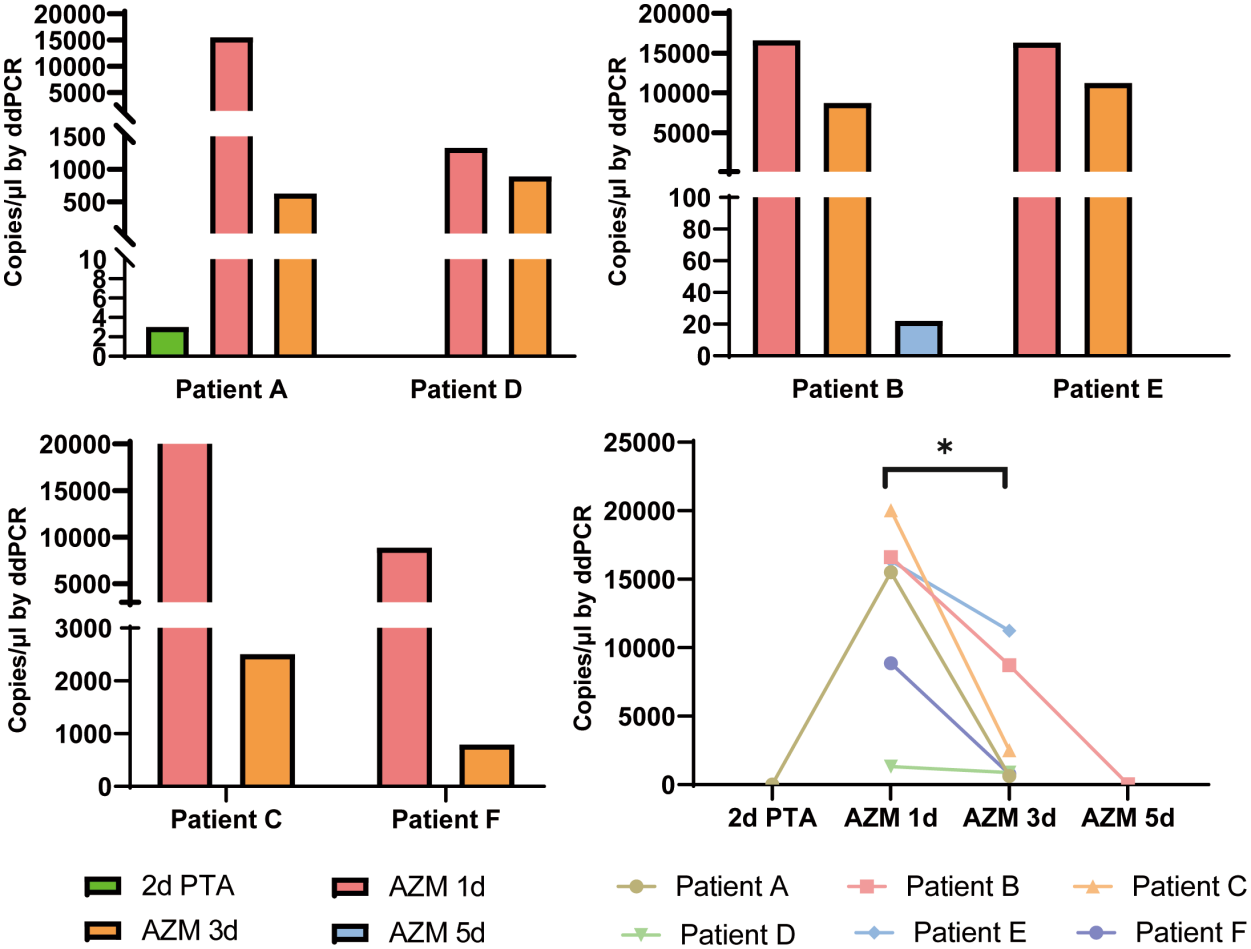
Dynamic changes of *M. pneumoniae* load in swab samples from six patients. The bacterial load numbers of six patients at different sample times were tested by ddPCR to reflect the clinical treatment efficacy. 2d PTA: 2 days prior to admission; AZM 1d: day 1 of azithromycin treatment; AZM 3d: day 3 of azithromycin treatment; AZM 5d: day 5 of azithromycin treatment. *p < 0.05.

In total, 178 clinical samples were tested for *M. pneumoniae* with ddPCR and real-time PCR. For ddPCR, 80 samples were positive and 98 were negative. For real-time PCR, 79 samples were positive and 99 were negative. The correlation coefficient between ddPCR and real-time PCR was 0.8784 (Figure 4A). There was one sample that showed a positive result with ddPCR and had three copy numbers but showed a negative real-time PCR result (Table 1). This sample was collected from patient A 2 days before admission, but the patient was classified as positive because the second sample was positive (data in Figure 5). The results showed that ddPCR and real-time PCR were accurate and reliable, but ddPCR was more accurate for samples with a low copy number.

**Table 1:** Performance of ddPCR and real-time PCR in clinical samples.

	Clinical Positive (80)	Clinical Negative (98)	Total (178)	PPV	NPV	Sensitivity	Specificity
ddPCR	80	98	178	100%	100%	100%	100%
qPCR	79	99	178	98.75%	100%	98.75%	100%

PPV, Positive predictive value; NPV, Negative predictive value.

### Bacterial load in children with different clinical features

Among the patients diagnosed with *M. pneumoniae* infection, 25 were classified into the SMPP group and 55 into the GMPP group based on the guidelines for management of pediatric community-acquired pneumonia. The duration of fever and length of stay were longer in the SMPP group than in the GMPP group (*p* < 0.001). White blood cell count, neutrophil ratio and C-reactive protein level were higher in the SMPP group than in the GMPP group (*p* < 0.001). The bacterial loads tested by ddPCR in the SMPP group were significantly higher than those in the GMPP group (*p* < 0.001, Figure 4B). The degree of bacterial load seemed to be related to the clinical features. Information on both groups is summarized in Table 2.

**Table 2:** Clinical information for children with *M. pneumoniae* pneumonia.

Parameters	General MPP	Severe MPP	*p* value
	(*n* = 55)	(*n* = 25)	
Sex/(Male count/percentages)	29 (52.72)	15 (60%)	0.158
Age (mean month, range)	63.4 (56.6–70.2)	70.5 (59.3–81.7)	0.0086
Duration of fever after recruitment (mean day, range)	9.13 (7.85–10.41)	14.1 (12.7–15.5)	< 0.001
Length of stay (mean day, range)	7.38 (6.56–8.10)	12.64 (10.18–15.1)	< 0.001
White Blood Cells (×10^9^ cells/L) (mean, range)	8.32 (7.43–9.21)	9.14 (8.59–9.69)	< 0.01
Neutrophils proportion (%) (mean, range)	58 (50–66)	65 (61–69)	< 0.001
C-reactive protein (mg/L) (mean, range)	18.3 (12.2–24.4)	41.6 (36.6–46.9)	< 0.001
Bacterial load by ddPCR (copies/μL) (mean ± SD)	2,973 ± 3,332	27,508 ± 15,946	< 0.001

### Dynamic changes in bacterial load in samples of six patients

Digital droplet PCR provided accurate detection of *M. pneumoniae*; therefore, we analyzed the dynamic changes in bacterial load throughout the treatment period. Six patients (patient C and E were classified as SMPP, the rest four patient were GMPP) with macrolide antibiotic treatment were sampled two or three times before or during admission. These additional eight samples were included for testing. Detailly, three swab samples which collected at 2 days prior to admission (2d PTA), day 1 of azithromycin treatment (AZM 1d), day 3 of azithromycin treatment (AZM 3d) were from Patient A. Three swab samples collected at AZM 1d, AZM 3d and day 5 of azithromycin treatment (AZM 5d) were from Patient B. For the rest four patient, two swab samples collected at AZM 1d and AZM 3d were collected separately. When tested by ddPCR, the bacterial loads of all patients dramatically decreased after azithromycin treatment (Figure 5, *p* = 0.014), which could have reflected the efficacy of antibiotic treatment. Patient A showed fluctuation in bacterial load, which initially increased and then decreased after macrolide treatment.

## Discussion

*M. pneumoniae* has substantially reduced its genome during its adaptive evolution, and thus it has limited metabolic capabilities and relies on its host to provide most of the nutrients it needs (Xiao et al., 2015). Hence, culturing this pathogen is difficult, and developing a fast and accurate alternative method for its clinical detection is important. Additionally, previous studies have confirmed that the severity of the clinical manifestations of respiratory infections is closely related to the copy number of the infecting bacterium or virus (Dorigo-Zetsma et al., 1999; Nilsson et al., 2010; Zhang et al., 2020); therefore, precise quantification of pathogen load is useful for assessing disease severity and progression.

Real-time PCR, a well-developed practical method for the detection of infective pathogens, has been widely used for the clinical diagnosis of *M. pneumoniae* (Dumke et al., 2007; Winchell and Mitchell, 2013; Zhao et al., 2020). However, it relies on a standard curve to calculate the DNA copy number, which differs among experiments and thus complicates the diagnostic process. Digital PCR, by contrast, quantifies nucleic acids independently of a standard curve and is less affected by PCR efficiency (Hindson et al., 2011; Li et al., 2018). Many studies have also proved that ddPCR shows better detection efficiency for samples with a lower copy number (Sedlak et al., 2017; Li et al., 2020), which may help improve the sensitivity of detection.

In this study, we developed a ddPCR assay for the detection and quantification of *M. pneumoniae*. A conserved region of the P1 gene was selected for the target because this region has been demonstrated to provide good sensitivity and specificity in PCR amplification (Waites et al., 2017; Leal et al., 2020; Xue et al., 2020). The other reported target genes included 16S rRNA, 16S-23S rRNA spacer, CARDS toxin gene, ATPase operon, dnaK, pdhA, tuf, parE, pdhA, ptsL, repMp1, and repMp4. The sensitivity and specificity of these targets differed. Our data suggested that ddPCR of P1 gene had high specificity with no cross-reaction to other respiratory and mollicute pathogens. When we tested the linear dynamic range and compared the results with those of real-time PCR, both methods showed excellent analytical performance. The LoD of ddPCR and real-time PCR was comparable; ddPCR was probably three times below that of the real-time PCR, which suggested that ddPCR was more suitable for detection of specimens with a low copy number, which is consistent with other studies (Jeremiah Matson et al., 2022; Milosevic et al., 2022).

In 178 patients, the results of real-time PCR and ddPCR were similar, except for one sample collected from patient A at 2 days before admission, in which the copy number detected by ddPCR was three, while that detected by qPCR was negative. Later, a second sample from this patient was positive by both methods. The two assays produced the same results for samples with a higher copy number. For patient A, even though ddPCR showed strong detection for the sample with a low copy number, we could not conclude that ddPCR was more sensitive than real-time PCR, as only one sample was tested. More samples are needed to verify the sensitivity of these two methods.

The clinical manifestations of *M. pneumoniae* infection differ among patients, and severe infection poses major therapeutic challenges (Izumikawa, 2016). At present, many research groups are exploring the clinical manifestations, laboratory examination results, and specific indicators of severe *M. pneumoniae* pneumonia. It has been shown that the load of *Mycoplasma* is positively correlated with disease severity (Wang et al., 2014). Therefore, accurate measurement of *Mycoplasma* load is important when determining disease severity, and digital PCR has advantages in this respect. In this study, we divided enrolled patients into two groups according to their clinical manifestations. After quantitative analysis of *Mycoplasma* from these patients, we found that the copy number of severe cases was generally >10^5^, which is consistent with other studies (Zhang et al., 2020). Zhang et al. divided patients into different groups based on copy number (10^5^–10^8^ copies) of *M. pneumoniae* genes determined using quantitative PCR, and showed that PCR loading was associated with clinical severity and inflammatory indicators. The high bacterial load group had high fever, high thermal peak, long duration of fever, and elevated levels of C-reactive protein. Many studies have also suggested that high macrolide resistance is related to clinical features (Zhou et al., 2021). In our study, total macrolide resistance mutations were found in 93.75% of samples, and there was no significant difference between the two groups (data not shown).

Dynamic changes to the bacterial or viral load during the course of infection are closely related to treatment efficacy. Recent studies have shown that SARS-CoV-2 load increases in the early and progressive stages and decreases in the recovery stage of COVID-19 (Yu et al., 2020; Leuzinger et al., 2021). *M. pneumoniae* is difficult to grow *in vitro*, and increasing reports of high rates of resistance to azithromycin heighten the significance of clinical bacterial load detection (Zhou et al., 2014). Because ddPCR can accurately detect pathogen loads in samples with a lower copy number, it is useful for evaluation of clinical treatment efficacy. Consistent with recent studies, the bacterial load of all six patients in this study decreased 3 days after macrolide treatment, even the specimens from all these six patients were macrolide resistance, but the trend differed among the six patients. Kawai et al. also found that in macrolide-resistant patients, some demonstrated a rapid decrease in the copy number of *M. pneumoniae* after 48 h of macrolide treatment, whereas others demonstrated no or only a slow decrease (Kawai et al., 2012). The high macrolide resistance in the present study indicated that the decrease copy number after treatment may have resulted from a combination of factors. The immunomodulatory effects of the macrolide therapy, and the host immune response are potentially involved. Utilization of macrolides in the presence of macrolide resistance mutations is controversial as to the effectiveness of therapy, which is not addressed in this study. Patient A was initially deemed negative for *M. pneumoniae* infection after real-time PCR, but a second sample 3 days later showed the patient to be positive. Which may because the first sample was collected at the initial stage of infection when the bacterial load was lower. When we tested the two samples by ddPCR, both samples were positive, but the first one showed only three copy numbers. This also demonstrated that ddPCR had higher sensitivity than real-time PCR.

Increasing test sensitivity, such as ddPCR, could help screen samples with low copy numbers, but that does not indicate true infection. Asymptomatic carriers of *M. pneumoniae* should also be considered (Spuesens et al., 2013). In that circumstance, the laboratory test results need to be confirmed with clinical symptoms, and resampling is necessary for diagnosis. If the patient is indeed infected, the copy number of the second sample usually increases with disease progression, as was the case in patient A in our study.

There were some limitations to this study. First, the clinical sample size was small. A larger size will give more accurate information, especially in terms of sensitivity, specificity and treatment efficiency. Second, only five *Mycoplasma* species were applied for specificity test. The other species which relevant to human disease should also be included. Last, different specimen types was related with bacterial load, only one sample type was collected from each patient, the difference between sample types was not observed in this study.

In conclusion, ddPCR was demonstrated to be a sensitive and accurate assay for *M. pneumoniae* detection, especially for low bacterial load samples, and showed a comparable detection efficiency to that of real-time PCR. ddPCR enabled absolute quantification of nucleic acids, without the need for a standard curve or reference sample each time; therefore, its performance in evaluating disease severity and treatment efficacy was excellent. However, ddPCR needs expensive equipment, complex operation, and experienced staff for evaluation of the results, which limit its widespread clinical application (Diaz and Winchell, 2016).

## Data availability statement

The original contributions presented in the study are included in the article/Supplementary material, further inquiries can be directed to the corresponding authors.

## Ethics statement

The studies involving human participants were reviewed and approved by the Ethics Committee of Capital Institute of Pediatrics. Written informed consent to participate in this study was provided by the participants’ legal guardian/next of kin.

## Author contributions

GX and JY designed and supervised the study. GX, HZ, and CY created the study methodology. HZ, CY, and YF collected clinical samples clinical data. HZ, JF, CY, BD, YF, XC, JC, LG, ZF, ZX, TF, and ZY processed samples and generated experimental data. GX, JY, HZ, and CY drafted and revised the manuscript. JY and GX obtained funding. All authors have confirmed that they have read and approved the final version of the manuscript.

## Funding

This work was supported by grants from the National Natural Science Foundation of China (32170201 and 82002191), National Natural Science Foundation for Key Programs of China Grants (82130065), Beijing Natural Science Foundation (7222014), FENG foundation (FFBR 202103), the Research Foundation of Capital Institute of Pediatrics (CXYJ-2021-04), and Beijing high-level public health technical talent project-02-08.

## Conflict of interest

The authors declare that the research was conducted in the absence of any commercial or financial relationships that could be construed as a potential conflict of interest.

## Publisher’s note

All claims expressed in this article are solely those of the authors and do not necessarily represent those of their affiliated organizations, or those of the publisher, the editors and the reviewers. Any product that may be evaluated in this article, or claim that may be made by its manufacturer, is not guaranteed or endorsed by the publisher.
