# Supplementary material for: Absolute quantification of Mycoplasma pneumoniae in infected patients by droplet digital PCR to track disease severity and treatment efficacy
Source: Front Microbiol. 2023 Jun 22;14:1177273. doi: 10.3389/fmicb.2023.1177273 (PMC10324665; doi:10.3389/fmicb.2023.1177273)
Supplement: Supplementary file 1 [file Table_1.DOCX]

**Table S1. Primers and probe for specific amplification of *M. pneumoniae***

| Name | Sequence (5′–3′) | Position in M129 |
| --- | --- | --- |
| P1-F | TCGTTGGTAGGGAACTCGTTTT | 182599-182600 |
| P1-R | GCTTTCAAGTTCATCGTACAGTAAGC | 182662-182687 |
| P1-probe | 6FAM- TACCATTACCATGGGTGATACCGCTACCG -BHQ1 | 182627-182655 |
